# Early T Cell Signalling Is Reversibly Altered in PD-1^+^ T Lymphocytes Infiltrating Human Tumors

**DOI:** 10.1371/journal.pone.0017621

**Published:** 2011-03-07

**Authors:** Shu-Fang Wang, Stéphane Fouquet, Maxime Chapon, Hélène Salmon, Fabienne Regnier, Karine Labroquère, Cécile Badoual, Diane Damotte, Pierre Validire, Eve Maubec, Nicolas B. Delongchamps, Aurélie Cazes, Laure Gibault, Marylène Garcette, Marie-Caroline Dieu-Nosjean, Marc Zerbib, Marie-Françoise Avril, Armelle Prévost-Blondel, Clotilde Randriamampita, Alain Trautmann, Nadège Bercovici

**Affiliations:** 1 Inserm, U1016, Institut Cochin, Paris, France; 2 Cnrs, UMR8104, Paris, France; 3 Univ Paris Descartes, Paris, France; 4 Inserm U970, Univ Paris Descartes, PARCC, Paris, France; 5 Service d'Anatomie-Pathologique, Hôpital Européen Georges Pompidou, APHP, Paris, France; 6 Laboratoire Microenvironnement immunitaire et tumeurs, INSERM U872, Centre de Recherche des Cordeliers, Paris, France; 7 Univ Pierre et Marie Curie, UMR S872, Paris, France; 8 Univ Paris Descartes, UMR S872, Paris, France; 9 Service d'Anatomie-Pathologie, Hôpital Hôtel Dieu, AP-HP, Paris, France; 10 Service d'Anatomie-Pathologie, Institut Mutualiste Montsouris, Paris, France; 11 APHP, UnivParis Diderot, Service de Dermatologie, Hôpital Bichat, Paris, France; 12 APHP, Hôpital Cochin, service d'Urologie, Paris, France; 13 Inserm U833, Collège de France, Université Paris Descartes, Paris, France; 14 Service d'anatomie et cytologie pathologiques, Groupe Hospitalier Cochin-Saint Vincent de Paul, Univ Paris Descartes, Paris, France; 15 APHP, Hôpital Cochin, Service de Dermatologie, Paris, France; Centre de Recherche Public de la Santé (CRP-Santé), Luxembourg

## Abstract

To improve cancer immunotherapy, a better understanding of the weak efficiency of tumor-infiltrating T lymphocytes (TIL) is necessary. We have analyzed the functional state of human TIL immediately after resection of three types of tumors (NSCLC, melanoma and RCC). Several signalling pathways (calcium, phosphorylation of ERK and Akt) and cytokine secretion are affected to different extents in TIL, and show a partial spontaneous recovery within a few hours in culture. The global result is an anergy that is quite distinct from clonal anergy induced *in vitro*, and closer to adaptive tolerance in mice. PD-1 (programmed death -1) is systematically expressed by TIL and may contribute to their anergy by its mere expression, and not only when it interacts with its ligands PD-L1 or PD-L2, which are not expressed by every tumor. Indeed, the TCR-induced calcium and ERK responses were reduced in peripheral blood T cells transfected with PD-1. Inhibition by sodium stibogluconate of the SHP-1 and SHP-2 phosphatases that associate with several inhibitory receptors including PD-1, relieves part of the anergy apparent in TIL or in PD-1-transfected T cells. This work highlights some of the molecular modifications contributing to functional defects of human TIL.

## Introduction

One of the generally accepted reasons for which the immune system has a low efficacy for fighting cancers is that tumor-infiltrating lymphocytes (TIL) are dysfunctional, because of an immmunosuppressive tumor microenvironment, and because TIL express inhibitory receptors, like PD-1. PD-1 is a receptor expressed by cells that are chronically activated, for instance in chronic viral diseases such as HIV, and it appears to contribute to the exhausted state of these cells [1]. Although these general notions are widely accepted, a closer look shows that the functional state of TIL remains ill-defined and would deserve a more stringent examination.

First, it is generally assumed that the functional state of TIL, corresponds to an alteration of early TCR signalling, as some key kinases (Lck, ZAP-70) have been described to be affected in such murine cells [2]. Some reports have mentioned the reduced expression of CD3ζ in T cells from cancer patients [3], [4]. However, this latter explanation cannot be a general one, as in several cancers including lung, CD3ζ expression was found to be normal [5], [6], [7]. The potential link between dysfunctions in early TCR signalling and expression of inhibitory receptor like PD-1 has not been addressed in fresh human TIL. Which are the early signalling pathways, such as calcium (Ca), ERK, Akt, that are affected in TIL, and to what extent inhibitory receptors are involved? In addition, in a number of studies, the functional state of TIL has been assessed after stimulating the cells with a combination of PMA and ionomycin. Such stimulation is informative on the differentiation state of the cells. However, it is inappropriate for evaluating the existence of alterations in early TCR signalling. For instance, both naïve and memory T cells show good responses in terms of early signalling with Ca or ERK as readouts; however, only effector/memory cells are able to secrete IFN-γ after stimulation by PMA/ionomycin, thus revealing their distinct differentiation state. Surprisingly, although several papers have shown that TIL are experienced T cells, as judged by their phenotype and their ability to respond to PMA/ionomycin [8], [9], others have described a failure to respond to these stimuli [10], [11].

A second problem relates to the T cell types that must be evaluated and compared, to understand T cell dysfunction in cancer. Although at late stages of the disease, a general defect of the immune system may be observed [4], at earlier stages, the function of anti-tumor specific T cells found in peripheral blood seems practically normal, and only TIL behaving abnormally [12]. It thus appears important to concentrate first on the analysis of defects observed in T cells found in the tumor microenvironment. But to which cells should TIL be compared? Peripheral blood T cells (PBT) constitute a first possible reference. If one wishes to compare TIL to T cells found in tissues, one should distinguish lymph nodes, visited by naïve and central memory T cells, and inflamed tissues, infiltrated by activated and memory T cells. In lymph nodes, the presence of chemokines [13], cytokines, and weak stimulation by self peptides on dendritic cells [14] results in a hypersensitivity of these cells to TCR stimulation, compared to the sensitivity of PBT. On the contrary, in inflamed tissues,the presence of a number of factors such as TGF-β results in an alteration of the sensitivity of these cells to TCR stimulation, compared to that of PBT [12]. In tumors, one expects to find a combination of immunosuppressive factors that are shared with non cancer inflamed tissues, together with factors that are tumor specific, including the presence of antigens able to elicit a chronic stimulation of TIL. Thus, there is no tissue that would constitute an obvious source of T cells to which TIL should be compared, and PBT appear to be a fair reference.

A third issue concerns the quantitative importance of PD-1 in contributing to the dysfunctional state of TIL, besides other, distinct immunosuppressive factors and its mode of action. The most common scheme is that PD-1 is an inhibitory receptor, able to recruit the phosphatases SHP-1 and SHP-2 [15], just like other inhibitory receptors such as KIR [16] expressed by NK and some T cells. The alterations of TCR signalling resulting from the presence of PD-1 are expected to resemble those resulting from the co-clustering of activation and inhibitory receptors in NK cells, leading to a blockade of the activation pathway. Even if such a scheme sounds plausible, it is far from having been demonstrated concerning the mode of action of PD-1 in human TIL. In particular, it assumes that an inhibitory effect of PD-1 can only be observed when it interacts with one of its ligands, PD-L1 or PD-L2, a point that deserves discussion. Indeed, some receptors have been shown to be active without engagement by their ligand [17]. Such a scheme should be tested by using early TCR signalling readouts. Instead, most conclusions concerning the importance of PD-1 in altering TIL functions have been based so far on late responses such as TIL proliferation that integrates many signals in addition to early TCR triggering. In addition, proliferation is measured after several days in culture, i.e., in cells that have been kept away from the tumor microenvironment. Even under these conditions, although the importance of PD-1 has been repeatedly stressed, and despite the existence of correlations between reduced patient survival and expression of PD-1 ligand (PD-L1) in human tumors [18], [19], a whole series of data shows that the real contribution to human TIL dysfunction could be quantitatively quite modest [20]. Finally, the commonly admitted explanation concerning the mode of action of PD-1 leads to the prediction that upon inhibition of SHP-1 and SHP-2, one should observe an improvement of early TCR signalling in TIL, which has never been demonstrated.

It thus appears that there is an important lack of precision and quantification in the common views concerning the characterization of early signalling defects in fresh human TIL, and concerning the relative contribution of PD-1 in TIL unresponsiveness. The present work aims at re-evaluating these different issues.

## Results

### Early T cell signalling are altered in human TIL

**Figure 1:**
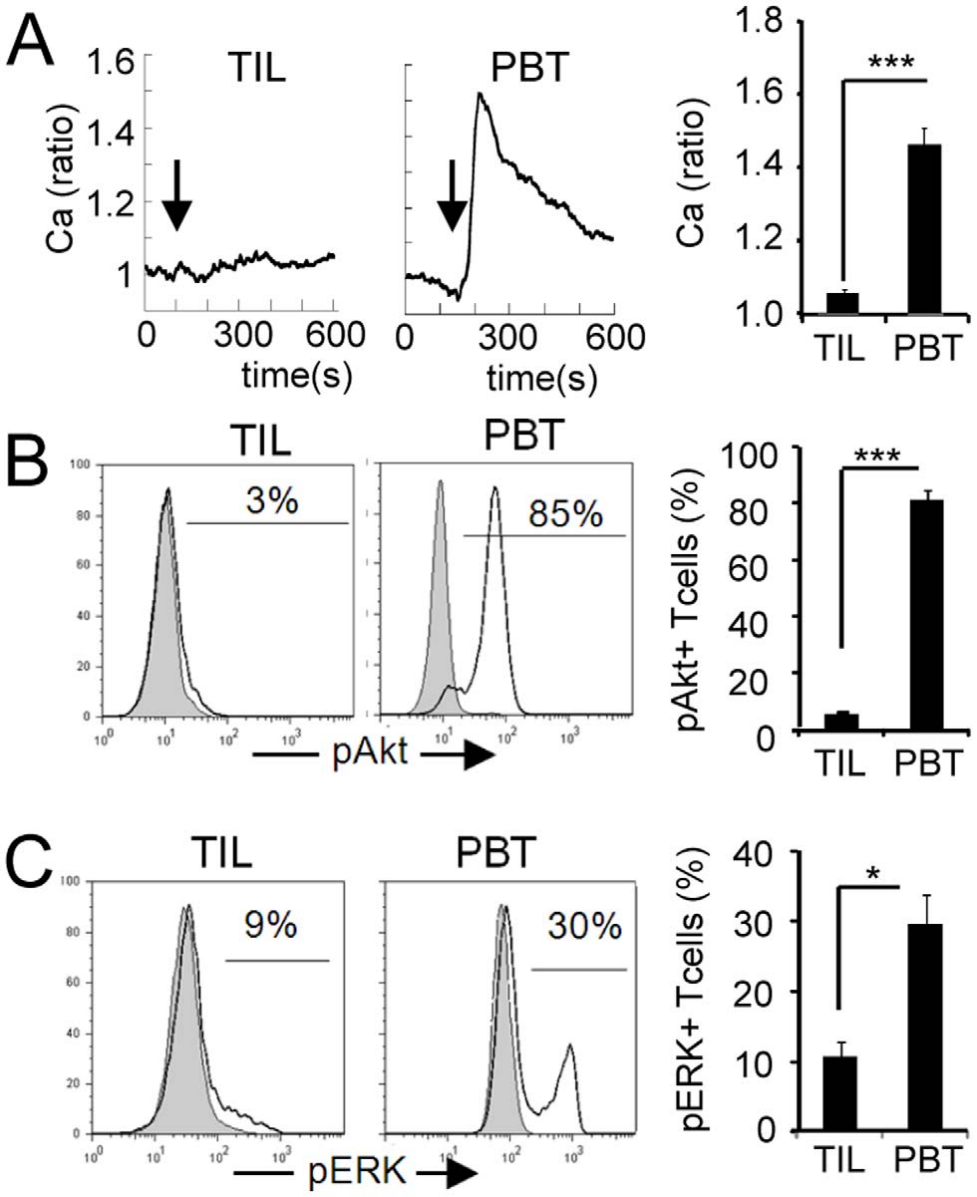
Early T cell signalling is altered in human TIL. (**A**) Left, typical example of Ca mobilisation after anti-CD3 stimulation (arrow) in TIL from a NSCLC biopsy, compared to PBT (In this and following figures, each Ca recording corresponds to the average of 10–30 cells). Right, mean Ca measured in TIL (NSCLC n = 23; melanoma n = 4; RCC n = 8) and PBT (n = 5 healthy donors). (**B**) Examples (left) and mean percentage (right) of Akt phosphorylation elicited by anti-CD3/anti-CD28 stimulation in TIL (n = 7 tumors) and PBT (n = 3 healthy donors). (**C**) Examples (left) and mean percentage (right) of anti-CD3-induced ERK phosphorylation in TIL (n = 12) and PBT (n = 3). Data represent mean +/− SEM; * p<0.05, ** p<0.01, *** p<0.001 (Student t test).

The functional state of human TIL freshly isolated from three types of tumors (non-small cell lung carcinoma (NSCLC), melanoma and renal cell carcinoma (RCC)) has been investigated within a few hours after tumor resection. Following TCR stimulation, the activation of the Ca, Akt and ERK pathways are required for a full activation [21]. Figure 1A shows that the Ca response elicited by anti-CD3 stimulation in TIL was severely depressed, on average ∼7 times smaller in TIL compared with peripheral blood T-cells (PBT). The phosphorylation of Akt induced by CD3/CD28 cross-linking was also severely affected (∼15 times reduction compared to PBT, Fig. 1B) whereas levels of phosphorylated ERK was only 2 to 3 times reduced in TIL after 2 minutes of anti-CD3-activation (Fig. 1C).

In addition, only a minority of TIL activated with anti-CD3/anti-CD28-coated beads produced IFN-γ (supplementary Fig. S1). Of note, PMA/ionomycin-activated TIL produced large amounts of IFN-γ, suggesting that the majority of TIL were differentiated in effector T cells capable of producing IFN-γ if early TCR signalling is bypassed.

Altogether, these data show that TIL have gone through a process of differentiation into effector cells, but that TCR signalling was blunted at an early level, affecting severely the Ca- and Akt-pathways, and to a lesser extent, ERK activation. These alterations led to a profound defect in the ability of TIL to produce IFN-γ in response to TCR stimulation.

### Upon interaction with myeloid or tumor cells, TIL give weak Ca responses

**Figure 2:**
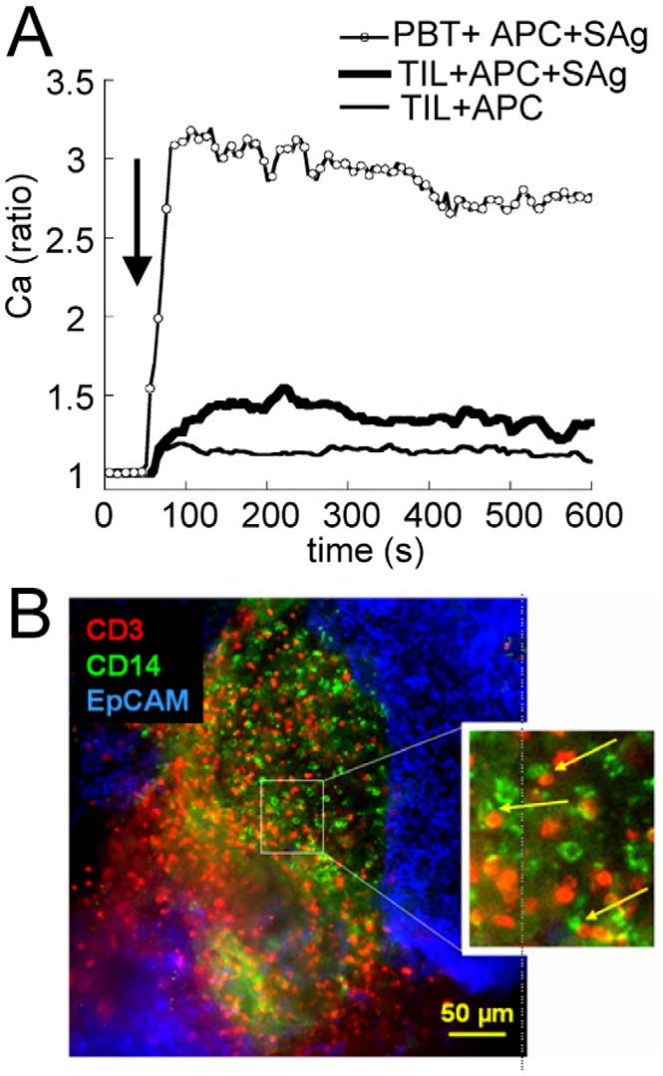
Interactions between TIL and large cells present in the tumor. (**A**) Average Ca responses triggered in TIL by interaction (arrow) with large cells either unloaded (thin line, average from 7 NSCLC tumors) or loaded with SAg (thick line, n = 6 NSCLC tumors) and in PBT (open symbols, n = 15 cells). (**B**) A typical triple labelling in a NSCLC slice, of TIL (CD3^+^ in red), monocytic (CD14^+^ cells in green) and tumor cells (EpCAM^+^ in blue). Zoom: arrows point to three CD3^+^-CD14^+^ conjugates.

Next, we examined if the weak reactivity of TIL was also observed upon interaction with freshly isolated tumor-derived cells presenting antigens. Such interactions led to occasional Ca responses of small amplitude (Fig. 2A). Even when pulsed with a cocktail of superantigens (SAg), tumor-derived cells triggered much smaller responses in TIL compared to PBT, indicating that TIL are dysfunctional even under conditions where antigen is not limiting. We then examined which cell type was the most likely to be in contact with TIL within the tumor. Observations of fluorescently labelled lung tumor slices indicate that TIL (CD3^+^ cells) do interact *in situ* with monocytes/macrophages (CD14^+^ cells) and are mostly present in the stromal zone (Fig. 2B) whereas the striking paucity of TIL in the tumor mass (EpCAM^+^) makes frequent TIL-tumor cells interactions quite unlikely. These important findings will be analyzed in detail in a distinct paper (Salmon et al, in preparation).

### TIL quickly recover part of their function after tumor dissociation

**Figure 3:**
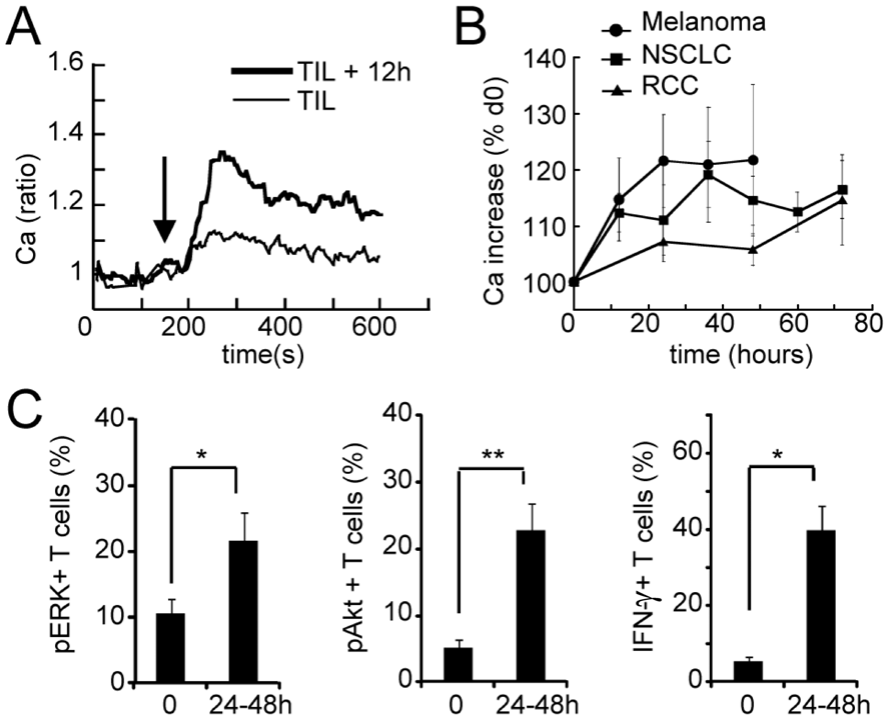
TIL show a spontaneous functional recovery in culture. (**A**) Typical average Ca responses in ex vivo NSCLC TIL and after 12 h in culture (n = 20–24 cells). (**B**) Average kinetics of Ca recovery in TIL from melanoma (n = 7), NSCLC (n = 23) and RCC (n = 7). (**C**) Percentage of phosphorylated ERK (left, n = 13), phosphorylated Akt (middle, n = 7) and IFN-γ producing TIL (right, n = 3), at day 0 and after 24–48 h in culture. Data represent mean +/− SEM ; * p<0.05, ** p<0.01, *** p<0.001 (Student t test).

After tumor dissociation, even though the different cell types present in the tumor remain in the same culture dish, the influence of the tumor microenvironment on the functional state of TIL may be reduced by the destruction of the native 3D structure. To examine the consequences of such an alteration of the microenvironment, we monitored the functional state of TIL in the hours following tumor dissociation. The ability of TIL to give a Ca response was markedly increased within a few hours (already at 12 h, Fig. 3A) with a plateau reached at 24 h and 36 h after dissociation of melanoma and lung tumors, respectively (Fig. 3B). The recovery of TIL from RCC was slow but was conspicuous at 72 h (Fig. 3B). Note that the addition of exogenous IL-2 in the culture did not improve the ability of TIL to recover a Ca response to anti-CD3 stimulation (data not shown). This differs from recovery of late effector TIL functions previously observed that required the presence of IL-2 [9], Thus, the disruption of the tumor microenvironment was sufficient to allow the recovery of a Ca response in TIL.

With lung tumors that allow the isolation of enough cells to study conjugates, Ca responses elicited by interaction between TIL and large cells could be observed (supplementary Movie S1) even in the absence of added superantigen, albeit larger in its presence. Under these conditions, we could detect a partial recovery of these responses, the average responses at 24 h being 136% and 112% of the responses *ex vivo* for conjugates formed with or without SAg, respectively (n = 3 tumors in each case).

The analysis of the functional recovery of TIL was further assessed by looking at the phosphorylation of ERK and Akt, and the ability to secrete IFN-γ. For all these readouts, a partial but significant recovery of TIL functions was observed after one to two days in culture (Fig. 3C).

Altogether, these data show that the functional alteration of TIL are reversible, at least in part, and that the severity with which the tumor microenvironment elicits TIL anergy depends on the tumor type.

### PD-1, but not its ligands, is frequently expressed in tumor biopsies

**Figure 4:**
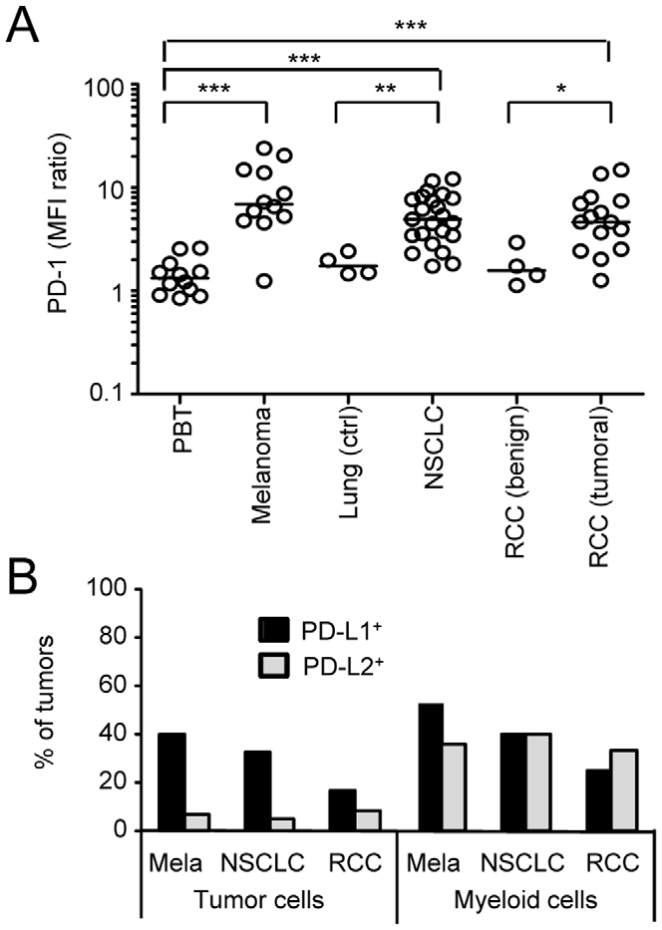
PD-1, but not its ligands, is frequently expressed in tumor biopsies. (**A**) PD-1 expression on PBT, melanoma, Lung specimens (control or tumoral) and RCC (benign and tumoral). PD-1 level is expressed as a ratio relative to the MFI of the control isotype. Bars correspond to the Median; * p<0.05, ** p<0.01, *** p<0.001 (Mann Whitney). (**B**) Percentage of tumor biopsies showing positivity for PD-L1 or PD-L2 staining (Melanoma n = 15; NSCLC n = 15; RCC n = 12).

As PD-1 expression on human TIL has been reported [22], [23], we examined how systematic this expression was, and to what extent it could contribute to TIL anergy. We show on three tumor types that a high expression of PD-1 is frequent on TIL (Fig. 4A and supplementary Fig. S2). In>90% of TIL from 50 tumors, the MFI ratio of PD-1, relative to isotype labelling, was >2, and in 68% of them >5. Much lower levels were observed on PBT or on T cells isolated from non-tumoral lung tissues or benign RCC.

The expression of the ligands of PD-1, PD-L1 and PD-L2, has been examined on tumor cells (CD45^-^) and myeloid cells (CD45^+^ ssc^high^ cells) freshly isolated from tumor biopsies. The levels of PD-L1 and PD-L2 were usually lower than those observed on activated monocytes (supplementary Fig. S2). Depending on the tumor type, PD-L1 was found to be expressed on tumor cells in only 18 to 40% of the biopsies (Fig. 4B). It was expressed at similar frequencies on myeloid cells infiltrating the tumors. PD-L2 was preferentially expressed on myeloid cells and was rarely detected on tumor cells. In rare cases (18% of tumor specimens), a very weak expression of PD-L1 was detectable on lymphocytes (data not shown) but it never reached the level observed on tumor or myeloid cells. The expression of PD-L1 was also detected by microscopy in some tumors but not others (Supplementary Fig. S3).

Thus, PD-L1 or PD-L2 are not expressed by every tumor, whereas a common feature between the different tumor types is the expression of PD-1 on TIL. One may thus wonder if PD-1 expression could influence TIL reactivity in tumors independently of engagement by its ligands.

### PD-1 expression is associated with a chronic activation and with the anergic state of TIL

Different approaches were used for assessing the functional importance of PD-1 in human TIL anergy.

First, we observed that TIL tended to lose PD-1 expression in culture, a roughly four-fold reduction being reached after 2 days (Supplementary Fig. S4A).

TIL maintained a significantly higher expression of PD-1 when cultured with anti-CD3 compared to non-stimulated TIL (*p*<0.05, n = 5, data not shown). In addition, PBT activated with anti-CD3/anti-CD28-coated beads express PD-1 and this expression was rapidly lost when the stimulation was interrupted, contrary to cells for which stimulation was sustained (Supplementary Fig. S4B), indicating that a continuous engagement of the TCR is necessary for maintaining a high PD-1 expression on T cells. These observations strongly suggest that TIL are chronically activated in the tumor microenvironment. This conclusion is further strengthened by the fact that 75% of TIL on average express CD69 *ex vivo* (data not shown).

**Figure 5:**
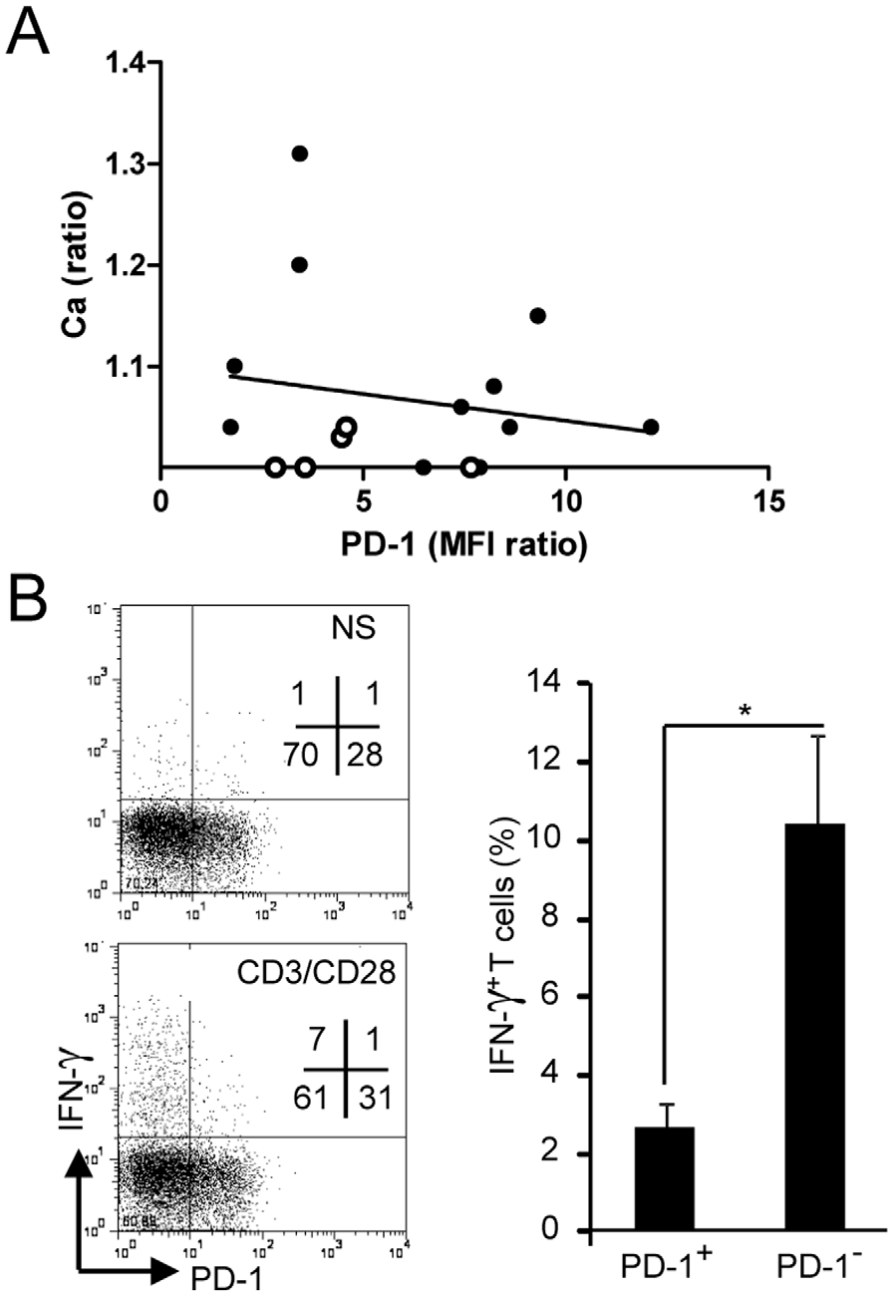
PD-1 contributes to the anergic state of TIL. (**A**) Ca responses and PD-1 levels measured on *ex vivo* TIL from 16 NSCLC. Open circles correspond to necrotic tumors. Linear regression curve is shown (r^2^ = 0.034). (**B**) Production of IFN-γ by TIL stimulated or not (NS) with anti-CD3/anti-CD28 coated beads. Left, typical dot plot from a NSCLC biopsy. Right, average (n = 3 tumors). Data represent mean +/− SEM; * p<0.05, ** p<0.01, *** p<0.001 (Student t test).

Next, we examined if there was a correlation between PD-1 expression and TIL reactivity *ex vivo* (Fig. 5A). Data were obtained with TIL from NSCLC, with which the largest number of experiments has been technically possible. At first glance, the anti-CD3-induced Ca response does not appear correlated with PD-1 expression by TIL. However, the largest Ca responses were never observed when TIL strongly expressed PD-1. A cluster of TIL shows a small Ca response despite the absence of strong PD-1 expression. Such a group may reveal that other inhibitory factors such as TGF-β and Galectin-3 play a key role in anergizing these TIL even when PD-1 is low [12], [24]. Interestingly, 4 out of the 6 tumors in the cluster with low PD-1 expression and Ca responses (white circles in Fig. 5A), showed a marked necrosis, an observation that might be related with the fact that TGF-β dependent healing mechanisms are triggered by cell death [25].

To further examine a possible link between PD-1 expression and TIL unresponsiveness, we looked at IFN-γ secretion by TIL. It clearly appeared that the few TIL able of producing IFN-γ *ex vivo* were PD-1^neg/low^, compared to PD-1^high^ TIL that do not produce any IFN-γ (Fig. 5B).

Altogether, these data indicate that the ability of fresh human TIL to respond to TCR stimulation is favoured by the low expression of PD-1.

### Sodium stibogluconate attenuates inhibitory signals in TIL, and in PBT overexpressing PD-1

**Figure 6:**
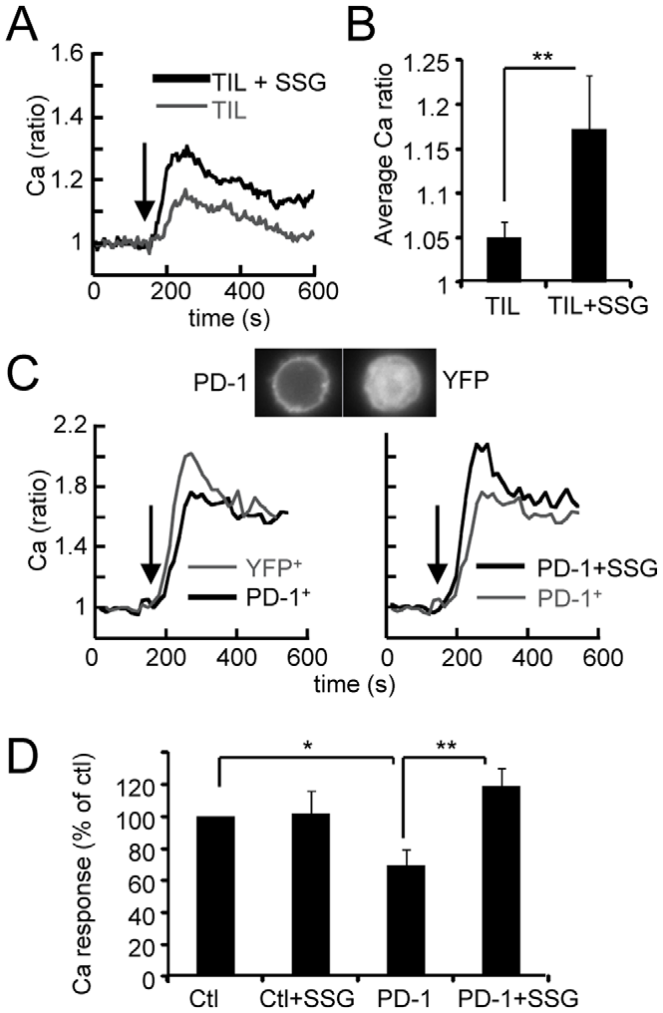
PD-1 expression delivers an inhibitory signal attenuated by sodium stibogluconate. (**A**) Typical average Ca responses elicited by anti-CD3 (arrow) in melanoma TIL, treated or not for 20 min with SSG. (**B**) Average Ca responses in TIL treated or not with SSG (n = 12 tumors). (**C**) Top: fluorescence images of PD-1 and YFP-transfected PBT. Bottom: typical average Ca responses triggered by anti-CD3 (arrows) in YFP^+^ and PD-1^+^ transfectants (left) and in PD1^+^ transfectants treated or not with SSG (right). (**D**) Average Ca response (in % of the response in YFP^+^ cells) in YFP^+^ and PD-1^+^ transfectants treated or not with SSG (n = 7 independent experiments). Data represent mean +/− SEM * p<0.05, ** p<0.01, *** p<0.001 (Student t test).

The intracellular part of PD-1 is able to recruit SHP-1 and SHP-2 after pervanadate treatment of T-cells, and a role of these phosphatases in PD-1 inhibitory signalling has been proposed [15]. When TIL were treated for 20 min with sodium stibogluconate (SSG), an inhibitor of SHP-1 and SHP-2 [26], the anti-CD3-induced Ca response of these cells was markedly increased, more than 3-fold on average (Fig. 6A and B). In addition, SSG-treated TIL tended to show larger Ca responses in conjugates with large cells freshly isolated from NSCLC as compared to untreated TIL (supplementary Fig. S5). We also examined if SSG could reverse the phosphorylation of ERK and Akt but we did not observe recovery for these pathways after a short treatment of TIL with the inhibitor (data not shown).

In all experiments performed with anti-CD3 stimulation, there was no concomitant engagement of PD-1 with its ligands, susceptible of providing an inhibitory signal. Is it conceivable that nevertheless, in such experiments, the mere expression of PD-1 was sufficient to deliver an inhibitory signal to T cells? To test this hypothesis, PBT were transfected with a full length human PD-1-YFP construct. In PD-1^+^ transfected-PBT, the anti-CD3-induced ERK response represented 74 +/− 7.5% (mean+/-SEM, n = 5) of that of YFP^+^ PBT. The anti-CD3-induced Ca response was also reduced and was 70+/−9.4% (n = 7) of that of YFP^+^ PBT (Fig. 6C). In addition, we could demonstrate that the inhibition of the Ca response (but not the ERK one), was fully reversed when PD-1^+^ PBT were treated with SSG (Fig. 6 C and D). Of interest, SSG had no effect on the anti-CD3-induced Ca response in control PBT (Fig. 6D). Thus, the mere expression of PD-1 is sufficient to partially inhibit anti-CD3-induced Ca and ERK responses, but only the inhibition of the Ca response appears to involve SSG-sensitive phosphatases.

## Discussion

In the present paper, we have characterized for the first time three signalling pathways that are simultaneously affected in human TIL, participating to their anergy. PD-1 expression contributes only to a fraction of this anergy. Unexpectedly, an inhibitory effect of PD-1 may be exerted even in the absence of its ligands. Finally, by using SSG, an inhibitor of SHP-1 and SHP-2, we show that these phosphatases contribute to the anergic state of TIL and to the ligand-independent inhibitory effect of PD-1.

Our data underline the importance of testing the functionality of TIL right after tumor resection, as the functional state of TIL monitored 12 h later is already different from that of freshly isolated TIL and thus, from TIL *in vivo*. In previous reports, the anergy of TIL was reversed by addition of IL-2 in culture [9] which was consistent with the view that experimental, ionomycin-induced anergy, could be reverted by adding exogenous IL-2. We have shown for the first time that a recovery of the Ca response of TIL may be observed a few hours after disrupting the tumor microenvironment, without the need to be supplemented with exogeneous IL-2.

By analyzing different pathways (Ca, Akt and ERK), we have identified several features of human TIL anergy. Our data show that this state differs from the existing murine models of anergy, in particular from a state extensively studied after treating T cells for 16 h with ionomycin [27], or after *in vitro* stimulation of the TCR without costimulation, a protocol called "signal 1 without signal 2". Indeed, experimental ionomycin-induced anergy affects preferentially the ERK pathway, whereas in real human TIL anergy, ERK is the least affected pathway. Human TIL anergy is more similar to the state of adaptive tolerance [28] triggered in murine models by chronic stimulation, and in which the Ca pathway is more severely affected than the ERK one. However, chronically stimulated murine "adapted" T cells do not express CD69, and express a modest level of PD-L1 [29], contrary to human TIL, that express CD69 but not PD-L1 (our data). Another difference between TIL and chronically stimulated murine "adapted" T cells is that TIL are usually in an inflammatory environment, including cytokines such as TGF-β, which is not necessarily the case in experimental models of chronic T cell stimulation.

PD-1 is a marker of recently activated T cells, which could be exhausted in some cases [1] and quite functional in other cases [30]. TIL are likely to be chronically activated cells, as judged by their expression of both CD69 and PD-1 and by the fact that PD-1 expression is maintained on TIL kept *in vitro* in the presence of anti-CD3 Ab, and decreases when the TCR signal is stopped. Although it is commonly considered that intratumoral TIL activation results from interactions with tumor cells, our data show that chronic TIL activation is more likely to result from interactions with cells present in the tumor stroma (in particular myeloid cells), because this is where TIL are mostly present. These chronically activated cells are experienced T cells, a conclusion that fits with our observation that they secrete cytokines in response to PMA/ionomycin, consistently with some reports [8], [9] but not all [10], [11]. Like memory cells, they have a lower CD28 expression than naïve T cells, which might make them less sensitive to anti-CD3/anti-CD28 stimulation than naïve T cells.

Our data show that PD-1 is not only a marker of TIL, it is also one of the factors that actively contribute to their anergy. This conclusion has already been proposed in several studies in which T cells were cultured for a week or so with anti-PDL1 antibodies [31], [32]. However, what was really tested in these studies was the ability of PD-1 to partially inhibit cell proliferation and associated cytokine production during *in vitro* recovery from anergy. For the first time, we have tested the functional state of *ex vivo* TIL with several readouts (Ca, phosphorylation of ERK and Akt) quite upstream TCR signalling, with no interference from the recovery phase. The responsiveness of these *ex vivo* TIL was shown to be favoured by the low expression of PD-1 (Fig. 5), although PD-1 was obviously not the only anergy-inducing molecule. The conclusion that PD-1 could interfere with TCR signalling was further strengthened by our observation that in PBT transfected with PD-1, a limited but significant inhibition of TCR signalling was observed. The possibility of an inhibitory effect of PD-1 even in the absence of ligand is of conceptual importance, as PD-1 ligands were not observed in all tumors. We propose that even in such tumors, PD-1 may still exert a partial inhibitory effect.

We have also shown that the Ca response of *ex vivo* TIL could be potentiated by SSG, an inhibitor of SHP-1 and SHP-2. This shows that an inhibitory signalling associated with these phosphatases contributes to TIL anergy. Such an effect does not allow distinguishing if these phosphatases are associated with PD-1 or with other inhibitory receptors. However, we have also shown that the inhibitory effect exerted by PD-1 in PD-1-transfected PBT was reversed by SSG, underlining the potential importance of the PD-1/SHP-1/SHP-2 pathway. This finding is of importance, given that SSG has already been successfully used to treat patients for another disease, leishmaniasis [33].

In conclusion, we have shown that several causes contribute to the weak antitumoral efficiency of TIL. First, as will be studied in more details in another study, their preferential location in the stroma rather than in contact with tumor cells does not favour an anti-tumor cytotoxic action. Second, TIL are in an anergic state that profoundly affects several signalling pathways, when analyzed immediately in *ex vivo* TIL, but which starts to reverse rapidly when the cells are put in culture. The systematic presence of PD-1 at the TIL surface is both a witness of their chronic activation in the tumor stroma, and one amongst several anergizing factors. The fact that a 20–30% inhibition of the anti-CD3-induced Ca response and ERK phosphorylations are observed in PBT transfected with PD-1 shows that PD-1 may exert an inhibitory effect even without interacting with its ligands. The observation that the Ca response of *ex vivo* TIL is more severely affected than that of PD-1-transfected PBT underlines that other causes concur to maintain anergy in TIL affected by a set of inhibitory mechanisms [12], [20], [24], [34]. The presence of two of them, membrane-associated TGF-β and Galectin-3 can be reduced by a short exposure of TIL to acidic pH ([12]; P. Van der Bruggen, personal communication). In our hands, such a treatment could indeed partially restore Ca response in fresh TIL (data not shown) without affecting PD-1 expression. This shows that even if TIL anergy cannot be only related to and correlated with PD-1 expression, PD-1 is one amongst several factors that all contribute to a fraction of TIL anergy. This study should help clarifying one of the aspects of the multifactorial phenomenon of human TIL anergy, which we hope to be able to reverse in the future.

## Materials and Methods

### Human Tumors and Ethics Statement

Fresh tumors were obtained with written informed consent prior to inclusion. Samples from patients with RCC (n = 18) and NSCLC (n = 40) were obtained from surgical therapeutic excision. Pathological examination of RCC tumors identified clear cell carcinoma (72%) and papillary carcinoma (28%). Four patients who were operated for benign renal tumors were also included as controls. NSCLC specimens were from adenocarcinoma (40%), large cell carcinoma (15%), squamous cell carcinoma (18%), and mixed histological features (18%). Lung biopsies (n = 4) were also collected from the surgical specimen at a distance from the primary NSCLC or from a different lesion-free lobe as tissue reference. Melanoma samples (n = 18) were collected from cutaneous metastasis who were removed for a pathological examination. The study protocol was approved by the CPP Ile de France ethical committee and was performed according to the Declaration of Helsinki Principles.

### Cell Isolation

Fresh biopsies were dissociated immediately after tumor resection. Single cell suspensions were prepared by mechanical dissociation followed by incubation in a non enzymatic solution (BD Cell recovery solution, BD Biosciences) for NSCLC (1 h at +4°C) or with collagenase D and DNAse enzymes (Roche) in RPMI without serum for cutaneous melanoma and RCC (30 min at 37°C). Cell suspensions were then filtered (100 µm) to remove membrane aggregates, rinsed twice with PBS 5% FCS 0.5 mM EDTA. TIL suspensions were enriched by Ficoll centrifugation (Ficoll-Paque Plus, Amersham Biosciences). For experiments with conjugates, TIL and large cells were separated on a Ficoll gradient. TIL were either used immediately or let to recover in short term culture at 2.10^5^ cells/200 µl in 96 U bottom well plates. IL-2 (10 U/ml) was added when TIL were used in cytokines detection assay after recovery. Peripheral blood T cells (PBT) from healthy donors (Etablissement Français du Sang, Paris) were isolated by Ficoll gradient followed by negative selection using a T cell isolation kit (BD Biosciences).

### PBT transfection with pEYFP-N1-PD1 construct

A full length human PD-1 cDNA (kindly provided by D. Olive, Marseille, France) was subcloned into pEYFP-N1 vector. PBT were nucleofected with PD-1 YFP or empty YFP vector (Amaxa), according to the manufacturer's instructions, and were used 18 h after transfection.

### Phenotyping by flow cytometry

Antibodies used for phenotyping PD-1 and its ligands (anti-CD3-PerCP, anti-PD1-PE, anti-CD45-FITC, anti-CD3-PerCP, anti-PDL1-PE, anti-PDL2-APC, anti-CD8-FITC, anti-CD4-APC, anti-CD69-PE Ab, IgG1-APC, IgG1-PE) were all from BD Pharmingen. Positive staining for PD-1, PD-L1 and PD-L2 were determined by comparing the MFI with specific mAb compared to the MFI with the respective control isotype. Cell suspensions were incubated for 30 min at +4°C with Ab, washed twice in PBS and fixed in PFA 1%. Cells were acquired on a FACSCalibur (BD) and analysed with FlowJo flow cytometry analysis Software.

### ERK and Akt phosphorylation

For ERK phosphorylation, washed TIL or PBT were stimulated with anti-CD3 Ab (10 µg*/*ml, UCHT1, BD Pharmingen) for 2 min at 37°C. For Akt phosphorylation, cells were coated with mouse anti-CD3 and mouse anti-CD28 (10 µg*/*ml, BD Pharmingen) Ab on ice for 20 min, followed by crosslinking with an anti-mouse IgG for 10 min at 37°C. After fixation with 4% PFA and permeabilisation with 0.1% saponin, cells were stained with purified mouse anti-human phosphorylated Akt (Cell signalling), phosphorylated ERK (Cell signalling) Ab and then with biotin conjugated donkey anti-mouse IgG Ab (Jackson ImmunoResearch Laboratories, INC.) and finally with streptavidin PE (BD Pharm). After washing, cells were acquired by FACSCalibur or fixed with 1% PFA and stored at +4°C until use.

### Calcium measurements

For imaging experiments, Ca responses were measured as previously described [35]. Briefly, T cells (PBT or TIL) were loaded with 0.5 µM Fura-2/AM (Molecular Probes) for 15 min at 37°C in mammalian saline. T cells were stimulated with 10 µg/ml anti-CD3 (UCHT1) or were added to tumor-derived large cells, pulsed or not with a cocktail of superantigens (SEA, SEB and SEE at 200 ng/ml, Toxin Technology). In some experiments, T cells were treated with sodium stibogluconate (SSG, 50 µg/ml, Calbiochem) during fura-2 loading, washed and maintained in medium containing SSG for the assay.

Image were acquired at 37°C every 5 s on a Nikon microscope (TE300 or TE2000), with a 10x or 20x objective. Cells were excited alternatively at 350 nm and 380 nm and emissions at 510 nm were used to measure Ca variations with Metafluor software (Molecular devices). In experiments with YFP-transfected PBT, YFP fluorescence was measured every 10 s in parallel to Ca. Ca levels are represented as a ratio 350/380 fluorescence intensity normalized to the ratio at t0.

For Ca measurements by flow cytometry, T cells were loaded with 1.5 µM Indo-1 for 30 min at 37°C, washed and rested for 10 before Ca measurements performed on a LSR2 (BD), the tube being maintained at 37°C during acquisition (360 nm excitation, 420 and 530 nm emission). The Ca baseline level was measured for 2 min before stimulation with anti-CD3 (UCHT1, 10 µg/ml).

### Intracellular cytokine staining

Cell suspensions (4.10^5^ cells/well) were incubated in 96 well U bottom plate with anti-CD3/anti-CD28 coated beads (1 bead for 5 cells, Dynal) or PMA (25 ng/ml) and Ionomycin (5 µg/ml) in the presence of Brefeldin A (2 µg/ml, Sigma). After overnight incubation, cells were washed in PBS 2% FCS and stained for detection of cell surface markers and intracellular cytokines with the IntraStain kit (BD) as recommended by the manufacturer. Cells were stained with anti-CD3-PerCP for 15 min at room temperature, fixed, permeabilized, and stained with anti- IFN-γ- APC Ab (BD Pharmingen) for 30 min at room temperature. After washing, cells were fixed with PFA 1% and stored at +4°C until acquisition.

### Immunofluorescence

Immunofluorescence on tumor slices was performed as described previously [36]. Briefly, small tumor pieces were embedded in 5% low-gelling-temperature agarose (type VII-1; Sigma-Aldrich). Slices (400 µm) were cut with a vibratome (VT 1000S; Leica) and fixed for 20 min with 2% PFA at room temperature. Immunostaining were performed at 4°C for 2 h or overnight with primary mAb specific for CD3 (IgG1, UCHT1), CD14 (IgG2a, BD Pharmingen) and biotinylated anti-EpCAM mAb (R&D System). Immunodetection was performed using Goat anti-IgG1 Alexa Fluor 647 and Goat anti-IgG2a Alexa Fluor 488 (Invitrogen) and Streptavidin-PE (BD Pharmingen), respectively. Antibodies (200 ng/slice) were diluted in PBS, 0,5% BSA, 2% AB serum. Images were obtained with an inverted microscope (TE2000-E; Nikon) equipped with a 20x and a 60x objective, and MetaVue imaging software.

### Cytokeratin/PD-L1 double staining of NSCLC frozen sections

Frozen pulmonary tissues were sectioned at 4–6 µm with a cryostat, placed on slides, air dried and fixed for 10 min with 100% acetone. Frozen tissues were stained with rabbit anti-human cytokeratin (Novocastra, Newcastle upon Tyne UK) and goat anti-human PD-L1 (R&D, Minneapolis, USA), followed donkey anti-rabbit biotinylated (Jackson Immunoresearch, West Grove, PA, USA) and FITC donkey anti-goat (Jackson Immunoresearch, West Grove, PA, USA) followed by cyanine 3-conjugated streptavidin (Amersham Biosciences, Uppsala, Sweden). Fluorescent images of mounted sections were analyzed with an epifluorescent microscope DMR (Leica Microsystems, Wetzlar, Germany).

## Supporting Information

Figure S1 Intracellular IFN-γ detection in NSCLC TIL stimulated or not (NS) with anti-CD3/anti-CD28 coated beads or with PMA/ionomycin. Left, typical dot plots. Right, average percentage of IFN-γ^+^ T cells (n = 3 NSCLC).
(TIF)

Figure S2 (**A**) Typical examples of PD-1 expression profiles on TIL from two different tumors (Tum1 and Tum2). (**B**) Example of PD-L1 and PD-L2 expression on CD45^-^ (tumor) cells and CD45^+^ ssc high (myeloid) cells from a fresh NSCLC biopsy, and on IFN-γ activated monocytes.
(TIF)

Figure S3 PD-L1 expression *in situ*. Top: example of NSCLC with PD-L1 expression; bottom: example of a negative tumor.
(TIF)

Figure S4 (**A**) Decrease of PD-1 expression in culture. PD-1 expression (MFI ratio) on TIL decreases in culture with time (n = 4 to 9 biopsies). Data represent mean +/− SEM. (**B**) PD-1 expression on day 6-activated T-cells decreases more rapidly in culture when TCR stimulation is stopped (no bead) compared to chronically activated T-cells (beads). Data represent mean +/− SEM from 3 independent experiments.
(TIF)

Figure S5 **Ca response in SSG-treated TIL conjugated with large cells from tumors.** Mean Ca responses measured in TIL treated (thick line, n = 3 NSCLC tumors) or not (thin line, n = 7 NSCLC) with SSG. Responses were triggered by the contact (arrow) with large cells isolated from the same tumors.
(TIF)

Movie S1 **Ca response in TIL interacting with a large cell isolated from a NSCLC tumor.** After 3 days in culture, fura-loaded TIL were added to large cells isolated from the autologous NSCLC tumor. Ca mobilisation in TIL was monitored under microscope as described in the Methods section. Both cell types are visible in the brightfield image (top); in the Ca movie (bottom), one can see that the TIL makes several Ca transients.
(AVI)
