# Supplementary figures and images for: Early T Cell Signalling Is Reversibly Altered in PD-1+ T Lymphocytes Infiltrating Human Tumors
Source: PLoS One. 2011 Mar 7;6(3):e17621. doi: 10.1371/journal.pone.0017621 (PMC3049782; doi:10.1371/journal.pone.0017621)

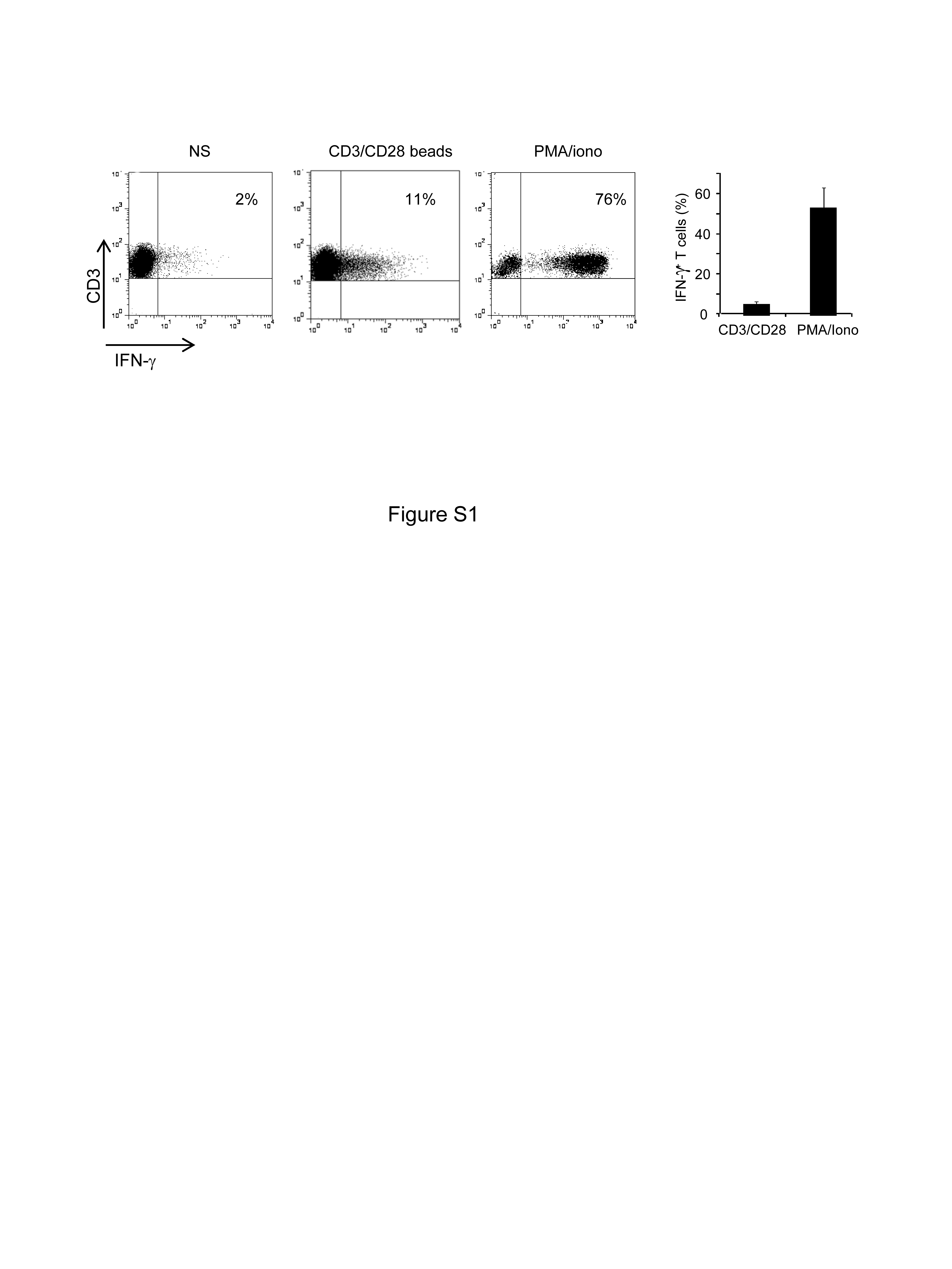

Supplement: Figure S1 — Intracellular IFN-γ detection in NSCLC TIL stimulated or not (NS) with anti-CD3/anti-CD28 coated beads or with PMA/ionomycin. Left, typical dot plots. Right, average percentage of IFN-γ+ T cells (n = 3 NSCLC). (TIF) [file pone.0017621.s001.tif]

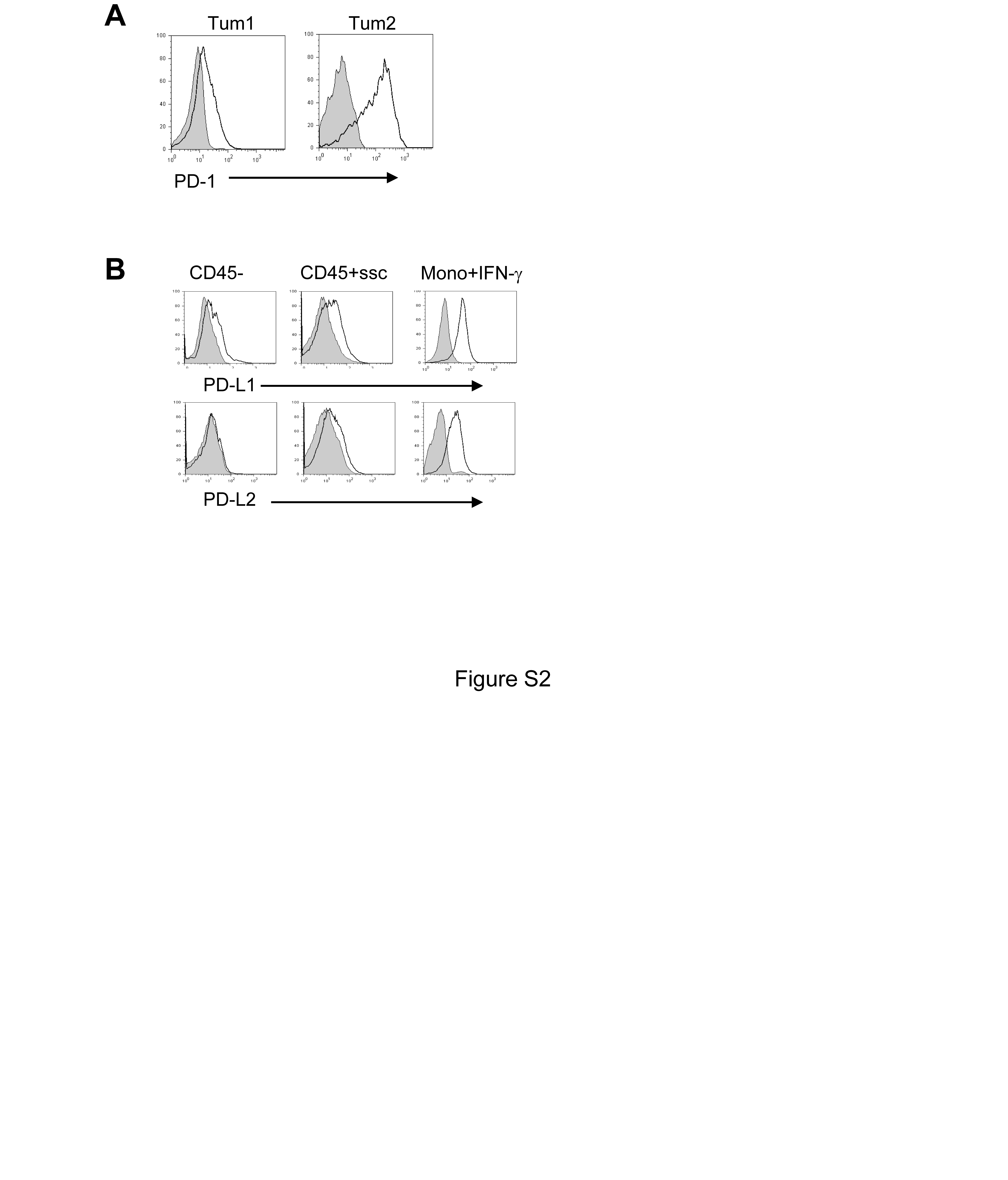

Supplement: Figure S2 — (A) Typical examples of PD-1 expression profiles on TIL from two different tumors (Tum1 and Tum2). (B) Example of PD-L1 and PD-L2 expression on CD45- (tumor) cells and CD45+ ssc high (myeloid) cells from a fresh NSCLC biopsy, and on IFN-γ activated monocytes. (TIF) [file pone.0017621.s002.tif]

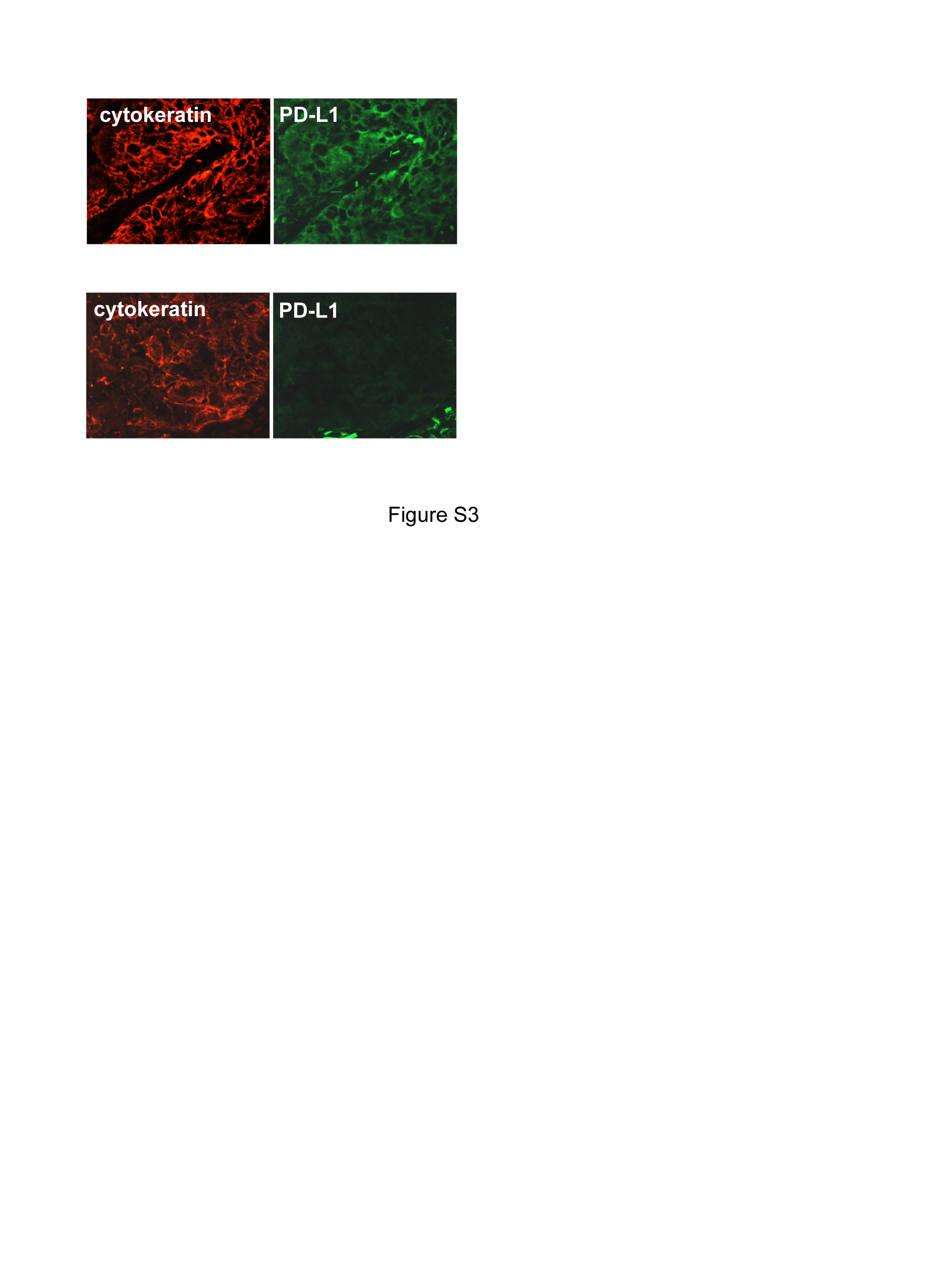

Supplement: Figure S3 — PD-L1 expression in situ. Top: example of NSCLC with PD-L1 expression; bottom: example of a negative tumor. (TIF) [file pone.0017621.s003.tif]

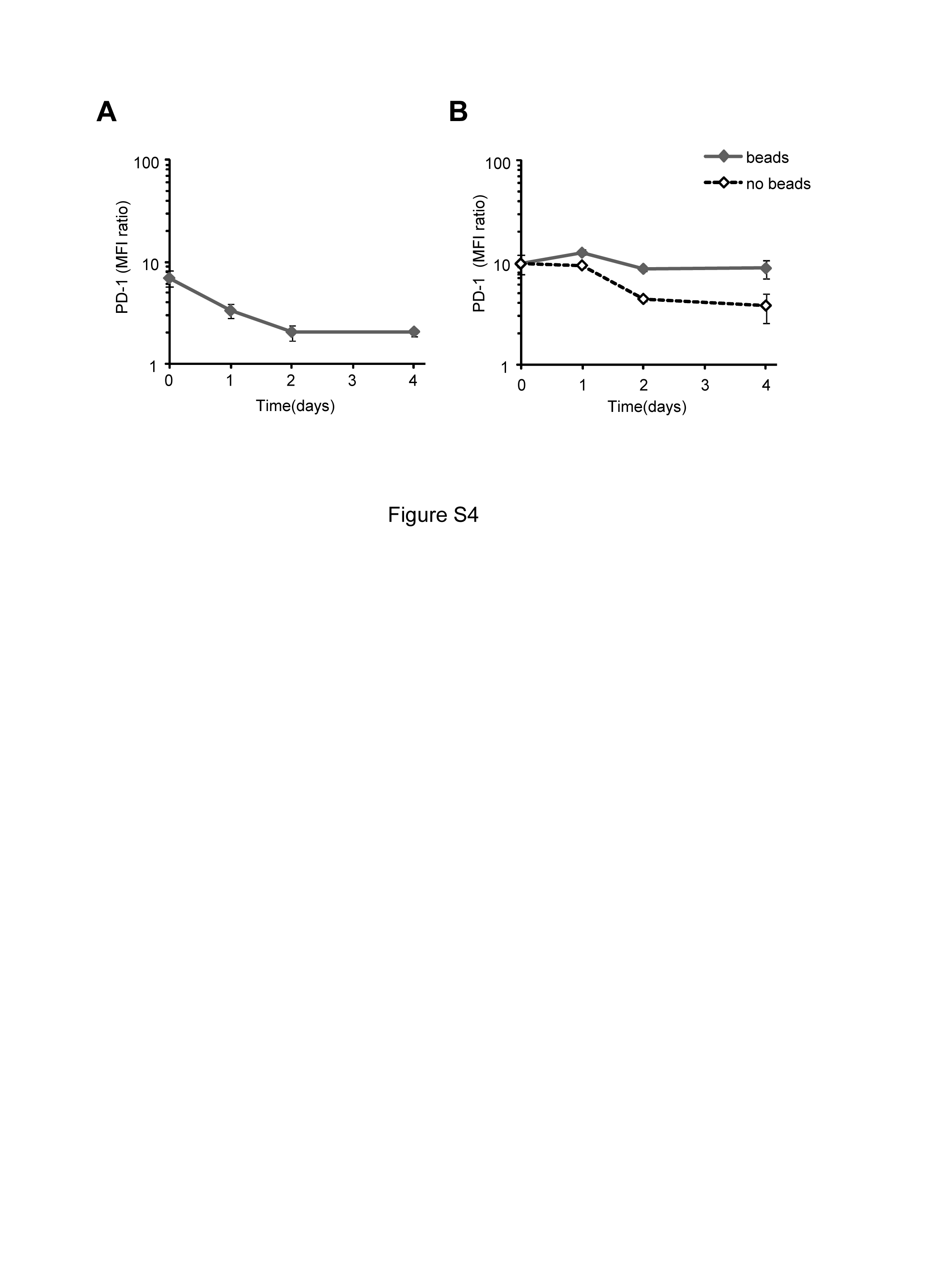

Supplement: Figure S4 — (A) Decrease of PD-1 expression in culture. PD-1 expression (MFI ratio) on TIL decreases in culture with time (n = 4 to 9 biopsies). Data represent mean +/− SEM. (B) PD-1 expression on day 6-activated T-cells decreases more rapidly in culture when TCR stimulation is stopped (no bead) compared to chronically activated T-cells (beads). Data represent mean +/− SEM from 3 independent experiments. (TIF) [file pone.0017621.s004.tif]

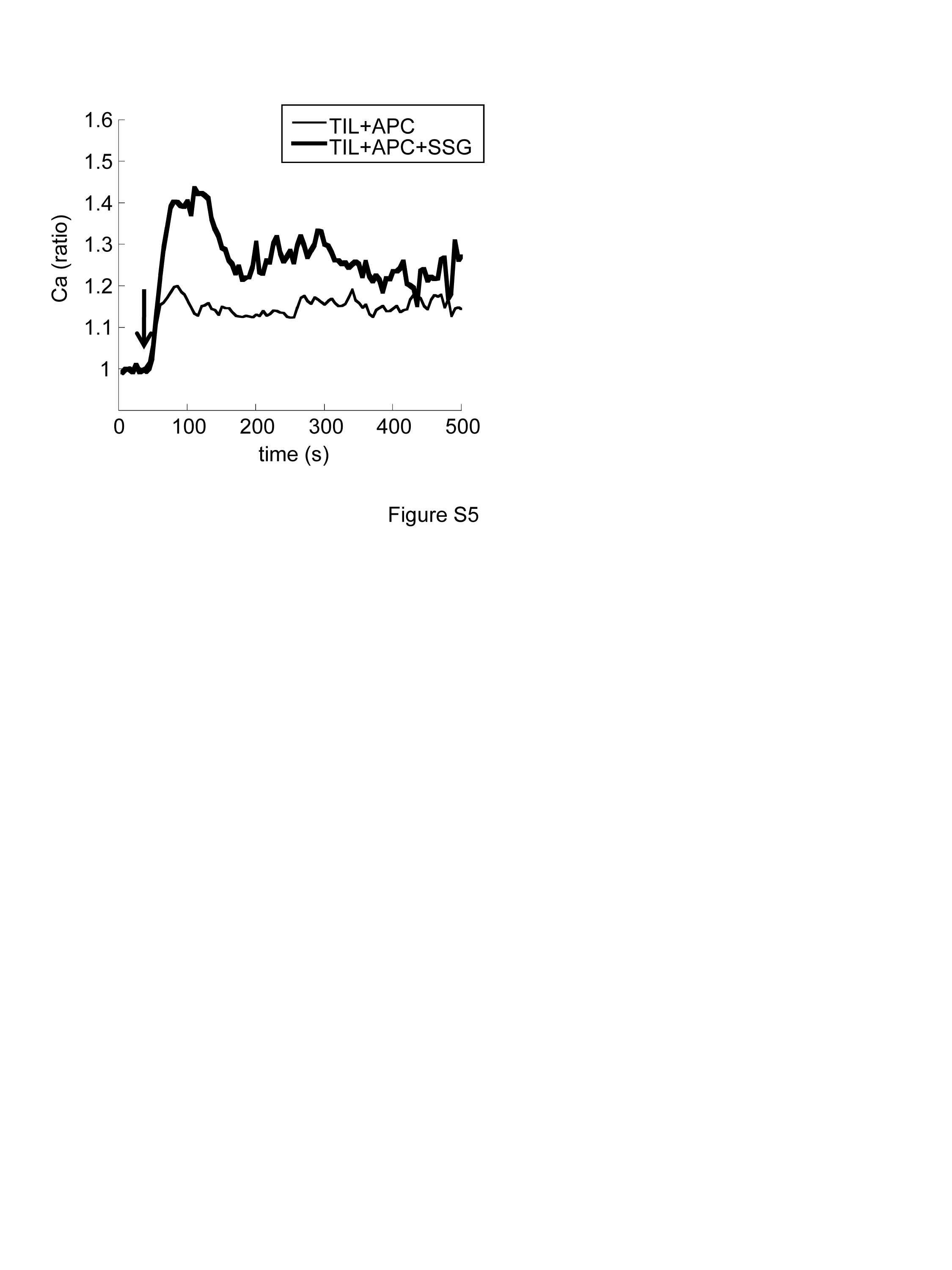

Supplement: Figure S5 — Ca response in SSG-treated TIL conjugated with large cells from tumors. Mean Ca responses measured in TIL treated (thick line, n = 3 NSCLC tumors) or not (thin line, n = 7 NSCLC) with SSG. Responses were triggered by the contact (arrow) with large cells isolated from the same tumors. (TIF) [file pone.0017621.s005.tif]
